# The role of ADAMTS‐13 and von Willebrand factor in cancer patients: Results from the Vienna Cancer and Thrombosis Study

**DOI:** 10.1002/rth2.12197

**Published:** 2019-05-06

**Authors:** Hanna L. Obermeier, Julia Riedl, Cihan Ay, Silvia Koder, Peter Quehenberger, Rupert Bartsch, Alexandra Kaider, Christoph C. Zielinski, Ingrid Pabinger

**Affiliations:** ^1^ Clinical Division of Hematology and Hemostaseology Department of Medicine I Medical University of Vienna Vienna Austria; ^2^ Department of Medical and Chemical Laboratory Diagnostics Medical University of Vienna Vienna Austria; ^3^ Clinical Division of Oncology Department of Medicine I Medical University of Vienna Vienna Austria; ^4^ Section for Clinical Biometrics Center for Medical Statistics, Informatics and Intelligent Systems Medical University of Vienna Vienna Austria

**Keywords:** ADAMTS‐13, neoplasms, thrombophilia, thrombosis, venous thromboembolism, von Willebrand factor

## Abstract

**Essentials**

- Cancer is associated with increased risk of developing venous thrombosis.
- Cancer patients were studied for ADAMTS‐13 and VWF levels and occurrence of venous thrombosis.
- Increased VWF in cancer patients is associated with a higher risk of venous thrombosis.
- Low levels of ADAMTS‐13 and/or increased VWF in cancer patients are associated with worse survival.

**Background:**

Cancer‐associated venous thromboembolism (VTE) is an important complication in the course of a malignant disease. Low ADAMTS‐13 (a disintegrin‐like and metalloproteinase with thrombospondin type 1 motif 13) and increased von Willebrand Factor (VWF) levels in cancer patients have been described numerously.

**Objectives:**

Investigation of the influence of ADAMTS‐13 and VWF on the probability of VTE and survival in malignancy.

**Patients/Methods:**

In the framework of the ongoing prospective Cancer and Thrombosis Study (CATS) ADAMTS‐13 activity and VWF antigen levels were investigated in cancer patients.

**Results:**

In total, 795 patients with various tumor types (364 female/431 male, median age 62 years) were included; of those, 56 developed VTE and 359 patients died during a median follow‐up time of 730 days. The hazard ratio (HR) of VTE per doubling of VWF level was 1.56 (95% confidence interval [CI] 1.13‐2.16) in multivariable competing risk analysis. ADAMTS‐13 levels showed no correlation with the incidence of VTE in univariate competing risk analysis. The HR of mortality per doubling of VWF level was 1.46 (95% CI 1.28‐1.66) and per SD increment of ADAMTS‐13was 0.90 (95% CI 0.81‐1.00) in multivariable Cox regression analysis. Patients with VWF >75th percentile and concomitant low (<25th percentile) or medium (25‐75th percentile) ADAMTS‐13 values had the highest probability of mortality (HR 4.31 and 4.75, respectively).

**Conclusions:**

High VWF levels were significantly associated with the risk of developing VTE in cancer patients, whereas ADAMTS‐13 was not. Low ADAMTS‐13 and increased VWF levels were independently associated with worse overall survival.

## 1 INTRODUCTION

Malignancy is an acquired hypercoaguable state with increased risk for venous thromboembolism (VTE).^1^ Cancer‐related VTE is associated with higher mortality^2^ and disease progression.^3^ In a recent Cochrane review it was discussed that thromboprophylaxis may potentially alleviate this frequent clinical problem, however, it comes with an increased risk of bleeding complications in cancer patients.^4^ Therefore, tailor‐made treatment based on reliable, individual risk stratification is required. Several risk factors that may contribute to the development of VTE in cancer patients have been described and different risk prediction scores were established.^5^

Von Willebrand factor (VWF) is a large polymeric glycoprotein involved in the adhesion and aggregation of platelets, particularly during vascular endothelial lesions. VWF is secreted into the plasma in the form of hemostatically highly active, “ultra‐large”‐VWF multimers (ULVWF).^6^ After conformational unfolding these multimers are cleaved by ADAMTS‐13 (a disintegrin‐like and metalloproteinase with thrombospondin type 1, motif 13).^7^ Cleavage results in smaller, less active VWF subunits. Complete deficiency in ADAMTS‐13 is the cause of thrombotic thrombocytopenic purpura (TTP), a thrombotic microangiopathy (TMA) whose hallmark symptoms are platelet‐ and VWF‐rich microvascular thrombi.^8^

Cancer patients have been shown to have higher VWF levels and lower ADAMTS‐13 levels than the general population,^9^, ^10^, ^11^, ^12^, ^13^, ^14^, ^15^, ^16^, ^17^, ^18^, ^19^, ^20^, ^21^ often also in a stage‐dependent intensity.^9^, ^10^, ^11^, ^12^, ^16^, ^20^, ^22^, ^23^, ^24^, ^25^, ^26^, ^27^, ^28^

In a recent study VWF and ADAMTS‐13 were shown to be associated with occurrence of VTE in cancer patients, with ADAMTS‐13 exhibiting predictive potential in risk scores.^21^ Patients with VTE or history of VTE have been repeatedly shown to have higher mean levels of VWF,^22^, ^23^, ^24^, ^25^, ^26^, ^27^ however, patients with underlying malignancy have always been excluded. Data on ADAMTS‐13 and its association with VTE, on the other hand, is ambiguous. There are studies showing decreased^28^ or increased^25^ ADAMTS‐13 activity with venous thrombosis. With next‐generation sequencing, one scientific group found an excess of rare coding single‐nucleotide variants of the ADAMTS‐13 gene in patients with deep vein thrombosis (DVT)^29^ while another group failed to find a link between DVT and a polymorphism associated with reduced levels of ADAMTS‐13.^26^

The interrelationship and calculated ratio between ADAMTS‐13 and VWF are also of particular interest. This topic has recently been studied in several settings and has been shown to be of potential use for predicting survival in patients with lung cancer^30^ or thrombotic complications in patients after hepatectomy.^31^

The aim of this investigation was to study the potential predictive value of ADAMTS‐13, VWF and their interrelationship for development of VTE in cancer patients and the correlation of values with survival probability in a large patient cohort.

## 2 METHODS

### 2.1 Study design

**Figure 1:**
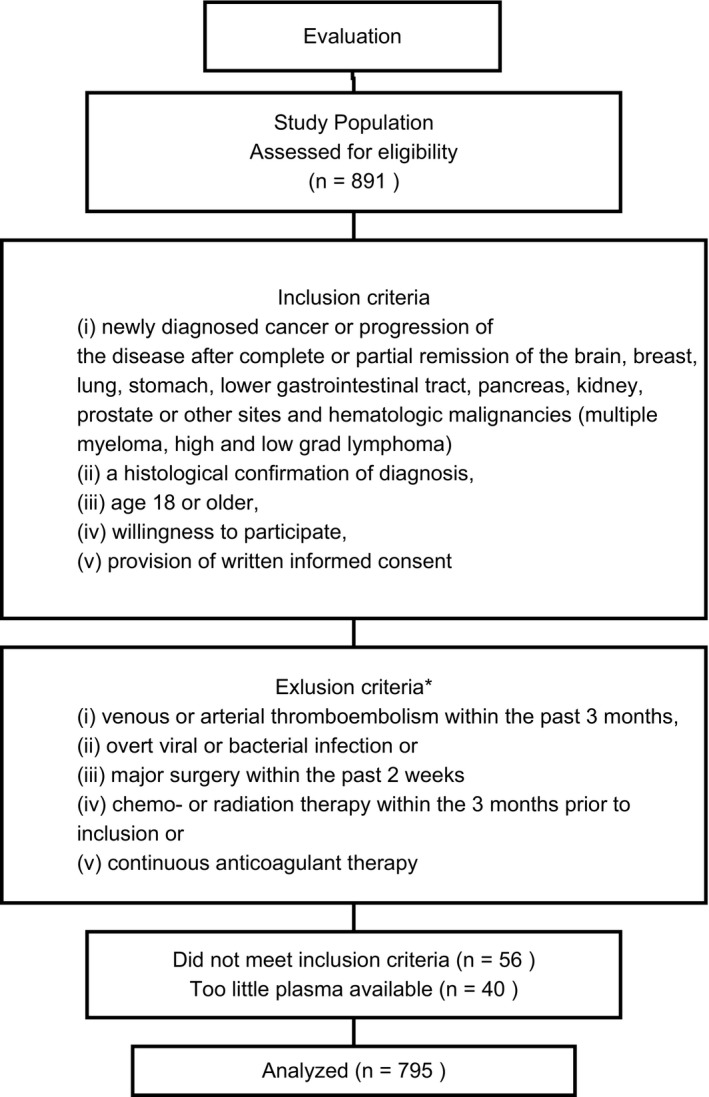
Consort diagram. *Antiplatelet therapy as well as temporary prophylactic treatment with low molecular weight heparin was accepted. Patients under prior or concomitant antineoplastic therapy were excluded for reason of possible transient effect on the coagulation system

The Vienna Cancer and Thrombosis Study (CATS) is an ongoing, prospective cohort study, approved by the local ethics committee and performed in accordance with the Declaration of Helsinki. It investigates potential predictive parameters for cancer‐related VTE. Cancer patients from different departments of the General Hospital of Vienna were included according to the criteria given in the consort diagram (Figure 1).

Study inclusion was performed via personal interview by a trained physician. Venous blood was drawn, mixed with one‐tenth volume sodium citrate stock solution to prevent clotting, centrifuged twice to obtain platelet‐free plasma (at 1500 *g* for 15 minutes and then 13 400 *g* for 2 minutes), frozen and stored at −80°C. Patients were educated on the possible signs and forms of presentation of VTE and advised to contact the study administration upon occurrence of any symptoms. Questionnaires regarding current medical status and possible VTE were sent by postal mail to patients in 3‐ to 4‐monthly intervals. If there was no response from the patient, information was sought by contacting family members, general practitioners or attending oncologists, and by annual check of the Austrian death registry regarding included study participants.

The study end point was an objectively confirmed VTE within a 2‐year observation period. Objective imaging methods to confirm VTE upon symptoms were Duplex sonography or venography for DVT or computed tomography or ventilation/perfusion lung scan for pulmonary embolism (PE). In patients that had died during follow‐up, death certificates and, if available, autopsy findings were reviewed to establish a diagnosis of fatal PE. All thrombotic events had to be confirmed by an adjudication committee, comprising independent specialists in angiology, radiology or nuclear medicine.

All authors had access to primary clinical data, which were analyzed by H.L.O, J.R., C.A., and I.P.

### 2.2 Laboratory measurement of ADAMTS‐13 and VWF

ADAMTS‐13 activity and VWF antigen were measured in the frozen patients’ plasma by a commercially available ELISA (Enzyme linked immunosorbent assay) kit (Technozym). Factor VIII (FVIII), D‐Dimer and soluble P‐selectin (sP‐selectin) were measured as previously described.^32^, ^33^, ^34^

### 2.3 Statistical analysis

Continuous variables were described with the median and the interquartile range (IQR). Categorical variables were described by the absolute number and percentages. Spearman correlation coefficient was used to describe the correlation between continuous variables.

Median follow‐up time was calculated by the reverse Kaplan‐Meier method.^35^ The analysis of variance models with the Tukey‐HSD test were applied to test for differences between tumor groups and stages.

Competing‐risk analysis for estimating the relative risk of VTE in the observation period was calculated according to the Fine and Gray proportional hazard subdistribution model.^36^ Within these regression models objectively confirmed VTEs were considered to be the event of interest, whereas deaths without developing VTE were treated as competing events and patients having reached the end of the observation period or being lost to follow‐up alive and without developing VTE were included as censored observations. As the amount of events limited the number of prognostic factors to be considered simultaneously, two multivariable models were designed. For the evaluation of the risk of VTE the first model included the following parameters: VWF, sex, age, and cancer type/stage. To consider tumor stage and site we categorized the patients into four groups according to Riedl et al^37^ In the second model values of VWF, D‐Dimer, sP‐selectin, and cancer type/stage were applied. Furthermore, possible time‐dependent effects of VWF and ADAMTS‐13/VWF were investigated within the univariate Fine and Gray models. ADAMTS‐13 was not considered in the multivariable competing risk model due to lack of correlation in univariate analysis and restriction of the amount of factors that could be applied.

To evaluate the influence on overall survival, three Cox regression models with the same parameters as above were applied; one for ADAMTS‐13 and VWF each and one with both factors combined. The event of interest was death of any cause and data were considered to be censored for patients having reached the end of the observation period or who were lost to follow‐up.

Because the distribution of values of VWF and ADAMTS‐13/VWF over the study population were skewed to the right, they needed logarithmic scaling to ensure correct statistical analysis. Values were scaled to the base of “2” which caused VWF and ADAMTS‐13/VWF to have the unit “per double increase” within the regression models by default. ADAMTS‐13 values, however, were distributed symmetrically, therefore the selected unit for ADAMTS‐13 values was a standard deviation (SD) increment.

To assess a possible non‐additive effect of ADAMTS‐13 and VWF on VTE and mortality, an interaction analysis was performed. Therefore, the product of ADAMTS‐13 and VWF was tested within the Competing risk model for VTE as well as in the Cox regression model for mortality, respectively.

For further investigation of the interaction of ADAMTS‐13 and VWF patients were categorized according to respective values <25th percentile, within the 25‐75th percentiles and >75th percentile. To illustrate additivity of ADAMTS‐13 and VWF in mortality, patients were divided into nine groups according to the respective category of both parameters. The HR for mortality within the nine different categories was calculated by the Cox regression model, adjusted for age, sex and cancer type/stage. For the illustration of VTE and survival probabilities, cumulative incidence curves, and Kaplan‐Meier plots were used.

To minimize potential bias laboratory analysis was blinded, which means that the persons who performed the laboratory analysis were not aware of the patients’ outcomes.

Two‐sided *P*‐values less than 0.05 were regarded as statistically significant.

Statistical analysis was performed with SAS 9.4 (SAS Institute Inc., Cary, North Carolina) and IBM SPSS Statistics 20.0 statistical software.

## 3 RESULTS

### 3.1 Characteristics of study participants

The total study population included 795 cancer patients; the basic demographic data are summarized in Table 1. Patients were followed up prospectively over a median observation period of 730 days (IQR 273‐731), 20 patients (2.5%) were lost for follow‐up.

**Table 1:** Basic characteristic of total study population, patients with VTE and deceased patients

Characteristic	Total study population (n = 795)	Patients with VTE^a^ (n = 56)	Patients that died (n = 359)
Median age	62	62	63
IQR	53‐68	53‐66	56‐70
Sex			
Female	364 (45.8)	23 (41.1)	152 (42.3)
Male	431 (54.2)	33 (58.9)	207 (57.7)
Site of cancer			
Brain	92 (11.6)	14 (25.0)	60 (16.7)
Breast	134 (16.9)	2 (3.6)	27 (7.5)
Lung	113 (14.2)	5 (8.9)	92 (25.6)
Stomach	36 (4.5)	6 (10.7)	26 (7.2)
Colorectal	108 (13.6)	8 (14.3)	47 (13.1)
Pancreas	46 (5.8)	6 (10.7)	37 (10.3)
Kidney	19 (2.4)	1 (1.8)	8 (2.2)
Prostate	94 (11.8)	2 (3.6)	18 (5.0)
Multiple myeloma	18 (2.3)	1 (1.8)	1 (0.3)
Lymphoma	89 (11.2)	7 (12.5)	17 (4.7)
Other	46 (5.8)	4 (7.1)	26 (7.2)
Classification of tumor^b^			
Localized	285 (35.8)	11 (19.6)	58 (16.2)
Distant metastasis	311 (39.1)	23 (41.1)	223 (62.1)
Not classifiable^c^	199 (25.0)	22 (39.3)	78 (21.7)
Newly diagnosed			
Yes	584 (73.5)	45 (80.4)	234 (65.2)
No	211 (26.5)	11 (19.6)	125 (34.8)
Died during observation period			
Yes	359 (45.2)	38 (67.9)	—
No	436 (54.8)	18 (32.1)	
Median survival in days	722	347	260
IQR	269‐731	155‐632	134‐439
ADAMTS‐13 (% of normal)	98.9	99.2	92.3
IQR	81.4‐115.9	83.7‐113.5	77.7‐111.1
VWF (IU/dL)	186	220	225
IQR	121‐272	138‐386	143‐357
ADAMTS‐13/VWF	0.53	0.46	0.42
IQR	0.31‐0.86	0.22‐0.72	0.22‐0.63
FVIII (% of normal)	179	196	197
IQR	137‐229	168‐248	159‐251
sP‐selectin (ng/mL)	42.9	47.4	44.0
IQR	33.8‐53.5	35.8‐62.7	34.2‐56.4
D‐Dimer (μg/mL)	0.71	0.89	0.97
IQR	0.36‐1.44	0.50‐2.18	0.59‐2.01

Note

Abbreviations: DVT, deep vein thrombosis; PE, pulmonary embolism; IQR, interquartile range; VWF, von Willebrand factor.

Categorical values are given as numbers and percentage in parenthesis and continuous values as median and IQR.

a The sites of thromboembolic events were as follows: 25 isolated DVTs of the lower extremity, 20 isolated PEs, three combined DVT and PE events, two fatal PEs and one case of portal‐, jugular‐, inferior cava‐ and sinus vein thrombosis, combined DVT of the lower extremity with portal vein thrombosis and PE combined with brachial vein thrombosis, respectively.

b Round‐off error for percentages in the first column.

c Patients with brain cancer, lymphoma and multiple myeloma

### 3.2 ADAMTS‐13 activity and VWF antigen levels

Median values for ADAMTS‐13 were lowest in lung and colorectal cancers but within normal range of 40%‐130% for most patients (Table 2). Only one patient with severe deficiency in ADAMTS‐13 (3% ADAMTS‐13 activity) was observed, another nine patients had moderately low levels(10%‐25%) and 25 patients had mildly reduced levels (25%‐40%), which together made up only 4.4% of the total study population. VWF values were highest in lung and pancreatic cancers. The ADAMTS‐13/VWF ratio was lowest in those with lung, colorectal and pancreatic cancers (Table 2).

**Table 2:** Medians and IQR of ADAMTS‐13, VWF, and the ADAMTS‐13/VWF ratio in the study population and various tumor types and stages

	n	ADAMTS‐13 (%)	IQR	VWF (IU/dL)	IQR	ADAMTS‐13/VWF	IQR
Study population	795	98.9	81.4‐115.9	186	121‐272	0.53	0.31‐0.86
Tumor type		†		‡		‡	
Brain	92	101.7	83.9‐116.3	206	120‐347	0.49	0.24‐0.83
Breast	134	103.8	86.5‐123.5	150	103‐226	0.69	0.39‐1.12
Lung	113	91.4	77.4‐106.0	212	140‐327	0.43	0.22‐0.64
Stomach	36	104.3	93.9‐119.3	203	137‐261	0.49	0.36‐0.80
Colorectal	108	92.2	74.6‐113.5	201	127‐281	0.44	0.27‐0.72
Pancreas	46	100.2	76.8‐114.0	233	148‐331	0.43	0.23‐0.62
Kidney	19	103.7	77.7‐120.8	153	119‐214	0.66	0.42‐0.93
Prostate	94	97.7	81.9‐117.0	140	111‐209	0.67	0.43‐1.01
Multiple myeloma	18	110.7	105.0‐123.7	163	127‐270	0.62	0.44‐1.01
Lymphoma	89	95.9	83.2‐110.1	151	116‐258	0.56	0.37‐0.83
Others	46	93.6	81.8‐116.0	210	138‐295	0.48	0.34‐0.66
Tumor stage^a^		†		‡		‡	
Localized	285	102.3	86.0‐120.7	158	110‐225	0.61	0.40‐0.98
Distant metastasis	311	92.0	77.3‐111.8	209	136‐325	0.44	0.25‐0.73

Abbreviations: IQR, interquartile range; VWF, von Willebrand factor.

^a^199 patients were not classifiable according to degree of metastasis (brain cancers, lymphomas and multiple myelomas).

*P <* *0.05, ^†^ *P *<* *0.01, ^‡^ *P *<* *0.001 for analysis of variance models for differences between tumor groups and stages.

ADAMTS‐13 and ADAMTS‐13/VWF ratio were significantly lower in metastatic compared to localized disease (*P *<* *0.01 and *P *<* *0.001, respectively). By contrast, VWF was significantly higher in patients with metastases compared to those without (*P *<* *0.001) (Table 2).

Very low ADAMTS‐13 values were not necessarily associated with very high VWF. There was only a weak correlation between ADAMTS‐13 and VWF (*r* = −0.126, *P *<* *0.001), whereas VWF had a strong correlation with FVIII (*r* = 0.65, *P *<* *0.001) and a positive correlation with D‐dimer (0.356, *P *<* *0.001).

### 3.3 Probability of VTE

In our patient cohort, VWF and the ADAMTS‐13/VWF were associated with occurrence of cancer‐associated thrombosis. In univariate competing risk analysis, a significantly increased risk for VTE was found for patients with levels of VWF > 75th percentile and ADAMTS‐13/VWF < 25th percentile, respectively. The hazard ratio (HR) per doubling of VWF level was 1.63 (95% confidence interval [CI] 1.23‐2.15). The HR per doubling of ADAMTS‐13/VWF ratio was 0.79 (95% CI 0.65‐0.96). No significant relationship between ADAMTS‐13 levels and risk of VTE could be found (HR per SD increment was 1.09 [95%CI 0.88‐1.35]).

**Figure 2:**
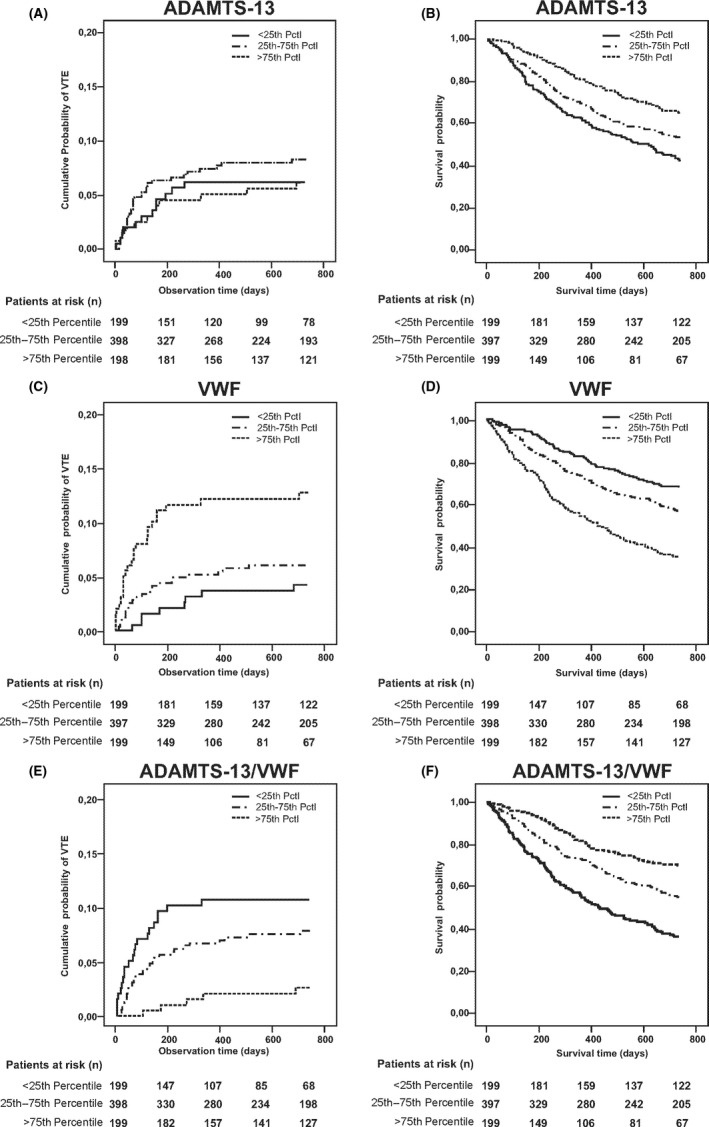
Cumulative incidence of VTE in competing risk analysis according to (A) ADAMTS‐13 values, (B) VWF values, and (C) ADAMTS‐13/VWF ratio classified into three groups of values above the 75th percentile, from the 25‐75th percentile, and below the 25th percentile. Patients at risk are given at 6‐mo intervals, observation period was 2 y (731 d). Patients with VWF levels >75th percentile and patients with ADAMTS‐13/VWF levels <25th percentile had higher probability of developing VTE. Difference in cumulative incidence was statistically significant for VWF and ADAMTS‐13/VWF (*P *<* *0.001 and *P *<* *0.05, respectively). No statistically significant association for levels of ADAMTS‐13 and VTE could be found. Kaplan‐Meier plots for survival probability of cancer patients according to (D) ADAMTS‐13 levels, (E) VWF levels, and (F) the ADAMTS‐13/VWF ratio classified into three groups of values above the 75th percentile, from the 25‐75th percentile and below the 25th percentile. Patients at risk are given at 6‐mo intervals, observation period was 2 y (731 d). Patients with higher ADAMTS‐13 and ADAMTS‐13/VWF and lower VWF levels had the best overall survival probability. Levels of ADAMTS‐13 and ADAMTS‐13/VWF <25th percentile or VWF >75th percentile were associated with worse survival. Differences in survival probability among groups compared with the logrank test were all highly statistically significant with *P *<* *0.001. VTE, venous thromboembolism; VWF, von Willebrand factor

Figure 2A‐C illustrates cumulative probability for developing VTE for all three variables. For patients with VWF values below the 25th percentile and above the 75th percentile after 2 years these were 4.2% and 12.8%, respectively. The corresponding probabilities in patients with ADAMTS‐13/VWF ratio above the 75th and below the 25th percentile were 2.6% versus 10.7%, respectively. As the flat, overlapping curves show, ADAMTS‐13 did not have a statistically relevant association with probability of VTE in cancer patients (Figure 2A).

There was a time‐dependent effect of VWF and ADAMTS‐13/VWF ratio on VTE probability. At baseline, HR per doubling of VWF level was 5.88 (95% CI 2.63‐13.2), while after 3 and 6 months it had gone down to 1.55 (95% CI 1.17‐2.06) and 1.26 (95% CI 0.87‐1.83), respectively. A similar pattern was seen with the ADAMTS‐13/VWF ratio with HR per doubling of ratio of 0.36 (95% CI 0.23‐0.58) at baseline, 0.83 (95% CI 0.68‐1.02) at three and 0.94 (95% CI 0.72‐1.23) at 6 months.

Results for VWF remained statistically significant for prediction of VTE in cancer patients after adjusting for possible confounding factors in two multivariable models (Table 3, Models 1 and 2). The HR per doubling of VWF level was 1.56 and 1.45, respectively (*P *<* *0.01 and *P *<* *0.05, respectively).

**Table 3:** Multivariable proportional hazard models for VTE and mortality

Patient group	Competing risk proportional hazard model for VTE				Multivariable Cox regression models for mortality					
	Model 1		Model 2		Model 3		Model 4		Model 5	
	HR	95% CI	HR	95% CI	HR	95% CI	HR	95% CI	HR	95% CI
ADAMTS‐13^a^	—	—	—	—	0.90*	0.81‐1.00	0.89*	0.81‐0.99	—	—
VWF^b^	1.56†	1.13‐2.16	1.45*	1.06‐2.00	1.46‡	1.28‐1.66	—	—	1.46‡	1.28‐1.66
Age	0.99	0.97‐1.02	—	—	1.01*	1.00‐1.02	1.02†	1.01‐1.03	1.01*	1.00‐1.02
Sex^c^	1.18	0.68‐2.03	—	—	1.11	0.89‐1.38	1.10	0.89‐1.36	1.16	0.94‐1.43
Cancer type	*		†		‡		‡		‡	
Brain	3.37†	1.43‐7.96	3.75†	1.64‐8.58	4.12‡	2.81‐6.05	4.98‡	3.42‐7.25	4.06‡	2.77‐5.95
Hematological	1.71	0.65‐4.51	1.83	0.73‐4.63	0.70	0.41‐1.19	0.76	0.44‐1.29	0.69	0.41‐1.18
Distant metastasis	1.56	0.72‐3.39	1.48	0.66‐3.29	3.96‡	2.93‐5.35	4.35‡	3.23‐5.87	3.99‡	2.96‐5.40
Localized	1		1		1	—	1	—	1	—
sP‐Selectin^b^	—	—	1.61*	1.00‐2.58	1.09	0.89‐1.32	1.07	0.88‐1.31	1.05	0.87‐1.28
D‐Dimer^b^	—	—	1.02	0.85‐1.21	1.24‡	1.15‐1.33	1.30‡	1.21‐1.40	1.25‡	1.16‐1.34

Abbreviations: CI, confidence interval; HR, hazard ratio; SD, standard deviation; VTE, venous thromboembolism.

^a^Per SD increment.

^b^Per doubling of level/value.

^c^HR of the female sex.

*P <* *0.05, ^†^ *P *<* *0.01, ^‡^ *P *<* *0.001.

### 3.4 Probability of survival

A statistically significant difference in survival probability according to values of ADAMTS‐13, VWF and the ADAMTS‐13/VWF ratio could be found in Kaplan‐Meier analysis (Figure 2D‐F). The probability of survival after 2 years in patients with VWF above the 75th and below the 25th percentile was 35.0% and 67.7%, respectively. The corresponding probabilities in patients with values >75th versus <25th percentile for ADAMTS‐13 were 65.3% versus 42.7% and for the ADAMTS‐13/VWF ratio 69.4% versus 36.1%, respectively. In univariate Cox regression analysis, the HR for mortality of ADAMTS‐13 activity per SD increment was 0.82 (95% CI 0.75‐0.91). The HR of per doubling of VWF level was 1.93 (95% CI 1.71‐2.18) and per doubling of ADAMTS‐13/VWF ratio was 0.64 (95% CI 0.59‐0.70).

In multivariable Cox regression analysis adjusting for possible confounding factors, both ADAMTS‐13 and VWF alone and in combination remained statistically significant for survival probability in cancer patients (both *P *<* *0.05 and *P *<* *0.001, respectively). Thus, both parameters serve as independent prognostic factors (Table 3, Models 3‐5).

### 3.5 Relationship between ADAMTS‐13 and VWF

It could be shown that ADAMTS‐13 and VWF do not interact with each other in our analyses. Investigation for a possible non‐additive effect of ADAMTS‐13 and VWF showed that the interaction term was not statistically significant for endpoints VTE (*P *=* *0.63) or mortality (*P *=* *0.36), neither in competing risk nor in Cox regression analysis. Therefore, it can be concluded these two factors contribute additively to our results.

**Figure 3:**
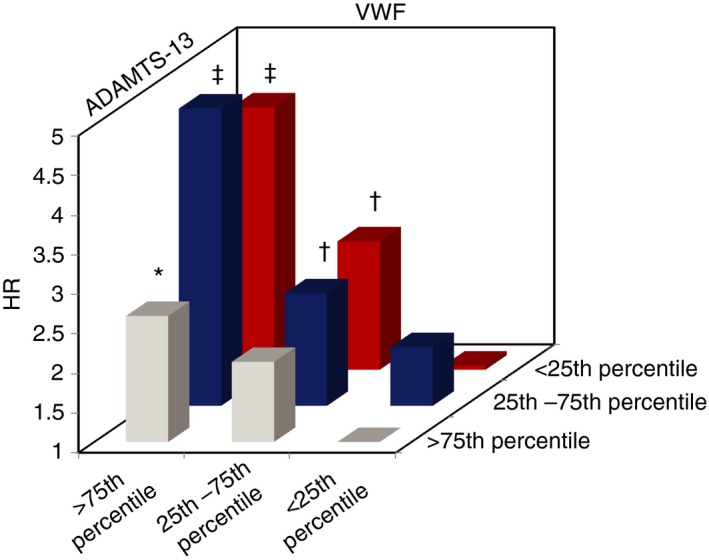
Hazard ratio for mortality according to the relationship between ADAMTS‐13 and VWF adjusted for categories age, sex, and cancer type/stage. Numerical data in Table 4, number of patients for respective categories in Table 5. Patients with values of VWF >75th percentile and concomitantly low (<25th percentile) or average (25‐75th percentile) ADAMTS‐13 values had the highest probability of mortality (HR 4.31 and 4.75, respectively). The negative impact of high VWF on survival probability seems most prominent, but a high ADAMTS‐13 also shows protective tendencies. Patients who had values of ADAMTS‐13 > 75th percentile and VWF <25th percentile and were used as reference (set as HR = 1). *P <* *0.05, ^†^ *P *<* *0.01, ^‡^ *P *<* *0.001. HR, hazard ratio; VWF, von Willebrand factor

To further illustrate additivity of both factors in a multivariable Cox regression analysis it could be demonstrated that cancer patients with VWF > 75th percentile and concomitantly low (<25th percentile) or medium (25‐75th percentile) ADAMTS‐13 values had the highest probability of mortality (HR 4.31 and 4.75, respectively) (Table 4). In relative numbers, within these two patient groups 81.5% and 68.5% of patients died within the observation period, respectively (Table 5) compared to 45.2% in the overall study population. The protective tendency of ADAMTS‐13 levels >75th percentile shown in univariate analysis can also be observed, however, it is seemingly not as pronounced as those of VWF levels <25th percentile (Table 4; Figure 3). When interpreting this data it has to be kept in mind that, because of the division of the patient cohort according to the levels of both factors, group sizes are not equally distributed. The absolute patient numbers are given in Table 5.

**Table 4:** Hazard ratio for mortality according to categories of ADAMTS‐13 and VWF values

	VWF >75th percentile	VWF 25‐75th percentile	VWF <25th percentile
ADAMTS‐13 <25th percentile	4.31‡ (2.15‐8.65)	2.62† (1.32‐5.20)	1.05 (0.41‐2.67)
ADAMTS‐13 25‐75th percentile	4.75‡ (2.42‐9.35)	2.42† (1.25‐4.69)	1.74 (0.85‐3.55)
ADAMTS‐13 >75th percentile	2.59* (1.20‐5.62)	2.01 (1.00‐4.04)	1

*Note* Categorization according to the following cut‐off values: ADAMTS‐13 < 25th and >75th percentile (81.4%‐115.9%, respectively) and VWF < 25th and >75th percentile (121‐272 IU/dL, respectively). Because of this categorization group sizes are not homogeneous (absolute numbers see Table 5). HR are adjusted for categories age, sex, and cancer type/stage. Values given as HR (95% CI). Patients with values of VWF < 25th percentile and ADAMTS‐13 > 75th percentile were used as reference (HR set as 1).

Abbreviations: CI, confidence interval; HR, hazard ratio; VWF, von Willebrand Factor.

*P <* *0.05, ^†^ *P *<* *0.01, ^‡^ *P *<* *0.001.

**Table 5:** Number of patients in the various categories and numbers patients who died according to ADAMTS‐13 and VWF values

Died/patients	VWF >75th percentile	VWF 25‐75th percentile	VWF <25th percentile
ADAMTS‐13 <25th percentile	53/65 (81.5)	50/101 (49.5)	8/32 (25.0)
ADAMTS‐13 25‐75th percentile	63/92 (68.5)	86/200 (43.0)	32/107 (29.9)
ADAMTS‐13 >75th percentile	19/39 (48.7)	38/101 (37.6)	10/58 (17.2)

*Note* Categorization according to the following cut‐off values: ADAMTS‐13 < 25th and >75th percentile (81.4%‐115.9%, respectively) and VWF < 25th and >75th percentile (121‐272 IU/dL, respectively). The number of patients who died and the number of patients in the according category. Percentage of patients who died within respective categories given in brackets.

Abbreviation: VWF, von Willebrand factor.

## 4 DISCUSSION

In the present cancer patient cohort a clear association between elevated levels of VWF and decreased levels of the ADAMTS‐13/VWF ratio and occurrence of VTE was found. In competing risk analysis patients with VWF values above the 75th percentile had a roughly three‐fold risk of developing VTE compared to those below the 25th percentile after 2 years. For the ADAMTS‐13/VWF ratio this difference was even four‐fold between levels <25th percentile and >75th percentile, respectively. VWF remained an independent predictor of VTE occurrence in cancer patients even after adjustment for patient‐ and cancer‐related factors in the first multivariable model or adjustment for previously validated biomarkers of cancer‐associated VTE^3^ in the second model. As the impact of VWF levels on risk of VTE was time‐dependent with the highest HR at baseline, its potential importance for prediction of cancer‐associated VTE at cancer diagnosis is emphasized. Overall, this may identify VWF as a possible laboratory tool for risk stratification of cancer patients prone to develop VTE. These results are in accordance with Pépin et al,^21^ who found statistically significant increased levels of VWF in cancer patients with VTE compared to cancer patients without VTE. Interestingly, in their patient cohort there was no significant difference in the levels of ADAMTS‐13 or ADAMTS13/VWF between cancer patients with VTE or without. However, they found the addition of ADAMTS‐13 to have added predictive value in risk assessment scores for cancer‐associated VTE. In our patient cohort we could not find any significant association in univariate competing risk analysis of only ADAMTS‐13 activity with VTE in cancer patients. However, patients with ADAMTS‐13/VWF levels >75th percentile had a cumulative probability of merely 2.6% of developing VTE after 2 years compared to 4.2% for patients with VWF <25th percentile (Figure 2D‐F). Therefore, it may be hypothesized that ADAMTS‐13 does influence the predictive potential of the ratio for cancer‐associated VTE, particularly concerning the stratification of low‐risk patients.

As a mechanistic explanation of this association it was hypothesized that cancer‐related VTE may be based on VWF‐mediated platelet aggregation.^38^ Bauer et al^39^ recently demonstrated in vitro that melanoma cells can activate vascular endothelial cells and prompt them to release ULVWF which is followed by platelet aggregation. They further showed that the combination of VWF release and decreased local ADAMTS‐13 in the tumor tissue is likely to cause a procoagulatory milieu. After infusion of recombinant ADAMTS‐13 (rADAMTS‐13) the formation of ULVWF networks and platelet aggregation was reduced. The potential benefit of rADAMTS‐13 for inhibiting thrombus growth and down‐regulating platelet adhesion to the endothelium was also established in other experimental studies.^40^, ^41^, ^42^ An approach via rADAMTS‐13 would be advantageous, as it cleaves only the highly active forms of the protein, leaving basal levels of VWF in the circulation.^41^ For a possible future outlook, in a recent review of therapies for TTP there was also promising data presented for both, rADAMTS‐13, which showed good tolerance in a phase 1 clinical trial, and for caplacizumab which is a nanobody that inhibits ULVWF mediated platelet aggregation under high shear rates,^43^ therefore theoretically also not interfering with basal VWF function.

One main point up for debate is the relationship and the (individual) cause and effect of either VWF and/or FVIII on thrombosis incidence, as one study found VWF to be an independent risk factor,^27^ whereas another found the correlation of VWF with VTE to be largely explained by FVIII in multivariable analysis.^23^ Both studies were performed in non‐cancer patients. VWF is the carrier molecule of FVIII in the circulation and also the main determinant of FVIII plasma levels.^44^ Because of the strong association between their values, we deliberately decided not to include FVIII in the multivariable models, as this causes both factors to mathematically nearly cancel out each other's predictive potential. Their close molecular relationship is also reflected by the strong correlation we found, so that a clear distinction regarding the individual cause and effect of VWF and FVIII on thrombosis incidence appears to be difficult at present. So the “chicken‐or‐egg causality dilemma” remains unanswered.

In the present cohort of patients with cancer ADAMTS‐13 was lowest in patients with lung, colorectal, and metastatic cancers but within the normal range in most individuals. Particularly high VWF levels were observed in pancreatic, lung, brain, stomach, and colorectal cancer patients and in those with distant metastases. This is in accordance with the literature, which frequently describes lower ADAMTS‐13 and higher VWF levels in cancer patients, often also in a stage‐dependent intensity.^9^, ^10^, ^11^, ^12^, ^16^, ^20^, ^22^, ^23^, ^24^, ^25^, ^26^, ^27^, ^28^

A stage‐dependent increase in VWF and/or respective decrease in ADAMTS‐13 could indirectly imply the correlation with worse survival of cancer patients. In the present study we could demonstrate that ADAMTS‐13 and VWF were associated with survival probability in patients with malignant disease. At the end of the observation period of 2 years, two‐thirds of patients with VWF values below the 25th percentile were still alive, but only one‐third of patients with VWF values above the 75th percentile. Similar data, but vice versa, were found for ADAMTS‐13 and ADAMTS‐13/VWF ratio. After adjustment in the multivariable models (Table 3) our results remained statistically significant, identifying both ADAMTS‐13 and VWF as independent predictors of survival probability (*P *<* *0.05 and *P *<* *0.001, respectively). This consolidated the literature data on an association of increased VWF and/or decreased ADAMTS‐13 with worse survival probability in colorectal cancer,^10^, ^45^ cancer of the head and neck,^46^ lung cancer,^30^ and in Waldenström's macroglobulinemia.^47^

There is, however, also an ongoing debate over the anti‐ or pro‐metastatic role of VWF. One study in VWF‐deficient mice showed an increase in metastatic foci of the lung, implying that VWF might be protective against tumor spread.^48^ On the other hand, another study could show in vivo that VWF fibers promoted the formation of lung metastases in the mouse model via the hematogenous route. Interestingly, in this study both VWF−/− and ADAMTS‐13−/− mice showed more metastatic lung foci. The authors hypothesize that VWF‐deficient mice come with further alteration of endothelial cell physiology (like missing Weibel palade bodies and dysregulated secretion of pro‐metastatic factors), which may be responsible for the increased metastatic potential.^38^

A main point of debate is whether the ADAMTS‐13/VWF ratio provides additional clinical information. Supposing that ADAMTS‐13 is the main determinant of VWF clearance,^22^ this suggests it is a principal regulator of VWF levels. The difference in the cumulative incidence of VTE after 2 years is greater when considering the levels of the ADAMTS‐13/VWF ratio than for VWF alone, thus possibly offering a better identification of patients at low risk of VTE. Furthermore, the HR for mortality of patients with high levels of VWF and concomitantly low or medium levels of ADAMTS‐13 was more than four‐fold compared to patients with high ADAMTS‐13 and concomitantly low VWF values (Table 4). Within the study population the median value of ADAMTS‐13/VWF was 0.53 (IQR 0.31‐0.86) while in those with VTE it was 0.46 (IQR 0.22‐0.72) (difference *P *<* *0.05). Results of the cohort of Pépin et al^21^ were similar, as cancer patients with VTE had mean ADAMTS‐13/VWF values of 0.36 (IQR 0.22‐0.49) and cancer patients without VTE had 0.44 (IQR 0.28‐0.57). A recent study showed that a ratio was superior for predicting mortality in patients with lung cancer than either parameter by itself.^30^ It can be hypothesized that in malignant disease a change in the proportional relationship can better reflect a disequilibrium of the two proteins most likely caused by a consumption of the protease due to an excess secretion of its substrate after endothelial activation. This concept has been described already for systemic inflammation and sepsis,^49^, ^50^, ^51^, ^52^, ^53^, ^54^ DIC,^55^, ^56^ severe dengue fever,^57^ and after the infusion of desmopressin.^58^ Further research is needed to either consolidate or reject the hypothesis that the ADAMTS‐13/VWF ratio is superior to either factor for predicting VTE and survival in cancer patients.

One limitation of our study was that only VWF antigen was tested, but not VWF activity. The amount of frozen plasma and the dimension of the study allowed for only one factor to be evaluated. However, in most cases VWF antigen and activity levels are highly correlated (*r* = 0.82), as recently demonstrated in a study with 432 patients, indicating the impact on the results are thus likely to be limited.^59^ Furthermore, patients were not tested for ADAMTS‐13 antibodies. Therefore, it cannot be excluded that the few patients with severe to moderate ADAMTS‐13 deficiency might have been positive for an inhibitor directed against the protease. Another limitation is the statistical modelling. Because of the comparably smaller amount of events in the competing risk analyses two separate models had to be devised. Also, in regard to the heterogeneity of our study population, cancer type and stage could not be evaluated in all subcategories. The design of dividing the models into four groups tries to address these limitations. The principal strength of our study, however, lies in the large number of diverse cancer patients studied for ADAMTS‐13 and VWF and in its long follow‐up period.

In conclusion, in this study we could demonstrate that levels of VWF antigen and ADAMTS‐13/VWF were strongly associated with the risk of VTE in cancer patients. In addition, a risk stratification model could lead to an improved sensitivity and specificity for prediction of VTE and thus allow a better tailoring of thromboprophylaxis to the individual of each patient. Moreover, a clear association of ADAMTS‐13 and VWF as well as of the ADAMTS‐13/VWF ratio with survival was found. These findings could have diagnostic, prognostic and—with further development of rADAMTS‐13—possibly also therapeutic implications in the future.

## RELATIONSHIP DISCLOSURE

The authors declare no competing financial interests.

## AUTHOR CONTRIBUTIONS

H.L.O, J.R., C.A, and I.P. prepared the manuscript, S.K and P.Q. were responsible for laboratory work, H.L.O. and A.K conducted the statistical analyses, I.P. C.A. and C.C.Z. were responsible for the study design and J.R. and R.B. recruited the patients.

## References

[1] Trousseau A . Phlegmasia alba dolens. Clin Medicale l'Hotel Dieu Paris. 1865;3:659–712.

[2] Levitan N , Dowlati A , Remick SC , Tahsildar HI , Sivinski LD , Beyth R , et al. Rates of initial and recurrent thromboembolic disease among patients with malignancy versus those without malignancy. Risk analysis using Medicare claims data. Medicine (Baltimore). 1999;78:285–91. 10499070 10.1097/00005792-199909000-00001

[3] Pabinger I , Thaler J , Ay C . Biomarkers for prediction of venous thromboembolism in cancer. Blood. 2013;122:2011–8. 23908470 10.1182/blood-2013-04-460147

[4] Di Nisio M , Porreca E , Candeloro M , De Tursi M , Russi I , Rutjes AW . Primary prophylaxis for venous thromboembolism in ambulatory cancer patients receiving chemotherapy. Cochrane Database Syst Rev. 2016;12:CD008500. 27906452 10.1002/14651858.CD008500.pub4 PMC6463937

[5] van Es N , Di Nisio M , Cesarman G , Kleinjan A , Otten HM , Mahé I , et al. Comparison of risk prediction scores for venous thromboembolism in cancer patients: a prospective cohort study. Haematologica. 2017;102:1494–501. 28550192 10.3324/haematol.2017.169060 PMC5685240

[6] Moake JL , Rudy CK , Troll JH , Weinstein MJ , Colannino NM , Azocar J , et al. Unusually large plasma factor VIII: von Willebrand factor multimers in chronic relapsing thrombotic thrombocytopenic purpura. N Engl J Med. 1982;307:1432–5. 6813740 10.1056/NEJM198212023072306

[7] Tsai HM . Physiologic cleavage of von Willebrand factor by a plasma protease is dependent on its conformation and requires calcium ion. Blood. 1996;87:4235–44. 8639782

[8] Asada Y , Sumiyoshi A , Hayashi T , Suzumiya J , Kaketani K . Immunohistochemistry of vascular lesion in thrombotic thrombocytopenic purpura, with special reference to factor VIII related antigen. Thromb Res. 1985;38:469–79. 2861671 10.1016/0049-3848(85)90180-x

[9] Koo BH , Oh D , Chung SY , Kim NK , Park S , Jang Y , et al. Deficiency of von Willebrand factor‐cleaving protease activity in the plasma of malignant patients. Thromb Res. 2002;105:471–6. 12091044 10.1016/s0049-3848(02)00053-1

[10] Wang WS , Lin JK , Lin TC , Chiou TJ , Liu JH , Yen CC , et al. Plasma von Willebrand factor level as a prognostic indicator of patients with metastatic colorectal carcinoma. World J Gastroenterol. 2005;11:2166–70. 15810086 10.3748/wjg.v11.i14.2166 PMC4305789

[11] Blann AD , Balakrishnan B , Shantsila E , Ryan P , Lip GY . Endothelial progenitor cells and circulating endothelial cells in early prostate cancer: a comparison with plasma vascular markers. Prostate. 2011;71:1047–53. 21557269 10.1002/pros.21319

[12] Blann AD , Gurney D , Wadley M , Bareford D , Stonelake P , Lip GY . Increased soluble P‐selectin in patients with haematological and breast cancer: a comparison with fibrinogen, plasminogen activator inhibitor and von Willebrand factor. Blood Coagul Fibrinolysis. 2001;12:43–50. 11229826 10.1097/00001721-200101000-00007

[13] Damin DC , Rosito MA , Gus P , Roisemberg I , Bandinelli E , Schwartsmann G . Von Willebrand factor in colorectal cancer. Int J Colorectal Dis. 2002;17:42–5. 12018453 10.1007/s003840100345

[14] Röhsig LM , Damin DC , Stefani SD , Castro CG , Roisenberg I , Schwartsmann G . von Willebrand factor antigen levels in plasma of patients with malignant breast disease. Braz J Med Biol Res. 2001;34:1125–9. 11514835 10.1590/s0100-879x2001000900004

[15] Böhm M , Betz C , Miesbach W , Krause M , Von Auer C , Geiger H , et al. The course of ADAMTS‐13 activity and inhibitor titre in the treatment of thrombotic thrombocytopenic purpura with plasma exchange and vincristine. Br J Haematol. 2005;129:644–52. 15916687 10.1111/j.1365-2141.2005.05512.x

[16] Fontana S , Gerritsen HE , Kremer Hovinga J , Furlan M , Lammle B . Microangiopathic haemolytic anaemia in metastasizing malignant tumours is not associated with a severe deficiency of the von Willebrand factor‐cleaving protease. Br J Haematol. 2001;113:100–2. 11328288 10.1046/j.1365-2141.2001.02704.x

[17] Oleksowicz L , Bhagwati N , DeLeon‐Fernandez M . Deficient activity of von Willebrand's factor‐cleaving protease in patients with disseminated malignancies. Cancer Res. 1999;59:2244–50. 10232615

[18] Weiss DR , Eiche C , Hupke C , Schellerer VS , Keller AK , Strasser EF , et al. The structure of the von Willebrand factor is not altered in patients with colorectal carcinoma. Colorectal Dis. 2012;14:1500–6. 22507880 10.1111/j.1463-1318.2012.03049.x

[19] Mannucci PM , Karimi M , Mosalaei A , Canciani MT , Peyvandi F . Patients with localized and disseminated tumors have reduced but measurable levels of ADAMTS‐13 (von Willebrand factor cleaving protease). Haematologica. 2003;88:454–8. 12681973

[20] Gadducci A , Baicchi U , Marrai R , Del Bravo B , Fosella PV , Facchini V . Pretreatment plasma levels of fibrinopeptide‐A (FPA), D‐dimer (DD), and von Willebrand factor (vWF) in patients with ovarian carcinoma. Gynecol Oncol. 1994;53:352–6. 8206409 10.1006/gyno.1994.1146

[21] Pépin M , Kleinjan A , Hajage D , Büller HR , Di Nisio M , Kamphuisen PW , et al. ADAMTS‐13 and von Willebrand factor predict venous thromboembolism in patients with cancer. J Thromb Haemost. 2016;14:306–15. 26589836 10.1111/jth.13205

[22] Nossent AY , VAN Marion V , VAN Tilburg NH , Rosendaal FR , Bertina RM , VAN Mourik JA , et al. von Willebrand factor and its propeptide: the influence of secretion and clearance on protein levels and the risk of venous thrombosis. J Thromb Haemost. 2006;4:2556–62. 17059421 10.1111/j.1538-7836.2006.02273.x

[23] Koster T , Blann AD , Briet E , Vandenbroucke JP , Rosendaal FR . Role of clotting factor VIII in effect of von Willebrand factor on occurrence of deep‐vein thrombosis. Lancet. 1995;345:152–5. 7823669 10.1016/s0140-6736(95)90166-3

[24] Bombeli T , Jutzi M , De Conno E , Seifert B , Fehr J . In patients with deep‐vein thrombosis elevated levels of factor VIII correlate only with von Willebrand factor but not other endothelial cell‐derived coagulation and fibrinolysis proteins. Blood Coagul Fibrinolysis. 2002;13:577–81. 12439142 10.1097/00001721-200210000-00001

[25] Mazetto BM , Orsi FL , Barnabe A , De Paula EV , Flores‐Nascimento MC , Annichino‐Bizzacchi JM . Increased ADAMTS13 activity in patients with venous thromboembolism. Thromb Res. 2012;130:889–93. 23031329 10.1016/j.thromres.2012.09.009

[26] Bittar LF , de Paula EV , Mello TB , Siqueira LH , Orsi FL , Annichino‐Bizzacchi JM . Polymorphisms and mutations in vWF and ADAMTS13 genes and their correlation with plasma levels of FVIII and vWF in patients with deep venous thrombosis. Clin Appl Thromb Hemost. 2011;17:514–8. 20682599 10.1177/1076029610375815

[27] Tsai AW , Cushman M , Rosamond WD , Heckbert SR , Tracy RP , Aleksic N , et al. Coagulation factors, inflammation markers, and venous thromboembolism: the longitudinal investigation of thromboembolism etiology (LITE). Am J Med. 2002;113:636–42. 12505113 10.1016/s0002-9343(02)01345-1

[28] Lancellotti S , Basso M , Veca V , Sacco M , Riccardi L , Pompili M , et al. Presence of portal vein thrombosis in liver cirrhosis is strongly associated with low levels of ADAMTS‐13: a pilot study. Intern Emerg Med. 2016;11:959–67. 27220954 10.1007/s11739-016-1467-x

[29] Lotta LA , Tuana G , Yu J , Martinelli I , Wang M , Yu F , et al. Next‐generation sequencing study finds an excess of rare, coding single‐nucleotide variants of ADAMTS13 in patients with deep vein thrombosis. J Thromb Haemost. 2013;11:1228–39. 23648131 10.1111/jth.12291

[30] Guo R , Yang J , Liu X , Wu J , Chen Y . Increased von Willebrand factor over decreased ADAMTS‐13 activity is associated with poor prognosis in patients with advanced non‐small‐cell lung cancer. J Clin Lab Anal. 2018;32:e22219 10.1002/jcla.22219. PMC6817161 28374895

[31] Kobayashi S , Yokoyama Y , Matsushita T , Kainuma M , Ebata T , Igami T , et al. Increased von Willebrand factor to ADAMTS13 ratio as a predictor of thrombotic complications following a major hepatectomy. Arch Surg. 2012;147:909–17. 23117828 10.1001/archsurg.2012.998

[32] Ay C , Simanek R , Vormittag R , Dunkler D , Alguel G , Koder S , et al. High plasma levels of soluble P‐selectin are predictive of venous thromboembolism in cancer patients: results from the Vienna Cancer and Thrombosis Study (CATS). Blood. 2008;112:2703–8. 18539899 10.1182/blood-2008-02-142422

[33] Ay C , Vormittag R , Dunkler D , Simanek R , Chiriac AL , Drach J , et al. D‐dimer and prothrombin fragment 1 + 2 predict venous thromboembolism in patients with cancer: results from the Vienna Cancer and Thrombosis Study. J Clin Oncol. 2009;27:4124–9. 19636003 10.1200/JCO.2008.21.7752

[34] Vormittag R , Simanek R , Ay C , Dunkler D , Quehenberger P , Marosi C , et al. High factor VIII levels independently predict venous thromboembolism in cancer patients: the cancer and thrombosis study. Arterioscler Thromb Vasc Biol. 2009;29:2176–81. 19778945 10.1161/ATVBAHA.109.190827

[35] Schemper M , Smith TL . A note on quantifying follow‐up in studies of failure time. Control Clin Trials. 1996;17:343–6. 8889347 10.1016/0197-2456(96)00075-x

[36] Fine JP , Gray RJ . A proportional hazards model for the subdistribution of a competing risk. J Am Stat Assoc. 1999;94:496–509.

[37] Riedl J , Kaider A , Reitter EM , Marosi C , Jager U , Schwarzinger I , et al. Association of mean platelet volume with risk of venous thromboembolism and mortality in patients with cancer. Results from the Vienna Cancer and Thrombosis Study (CATS). Thromb Haemost. 2014;111:670–8. 24306221 10.1160/TH13-07-0603

[38] Goertz L , Schneider SW , Desch A , Mayer FT , Koett J , Nowak K , et al. Heparins that block VEGF‐A‐mediated von Willebrand factor fiber generation are potent inhibitors of hematogenous but not lymphatic metastasis. Oncotarget. 2016;7:68527–45. 27602496 10.18632/oncotarget.11832 PMC5356571

[39] Bauer AT , Suckau J , Frank K , Desch A , Goertz L , Wagner AH , et al. Von Willebrand factor fibers promote cancer‐associated platelet aggregation in malignant melanoma of mice and humans. Blood. 2015;125:3153–63. 25977583 10.1182/blood-2014-08-595686 PMC4432010

[40] Chauhan AK , Motto DG , Lamb CB , Bergmeier W , Dockal M , Plaimauer B , et al. Systemic antithrombotic effects of ADAMTS13. J Exp Med. 2006;203:767–76. 16533881 10.1084/jem.20051732 PMC2118248

[41] Bergmeier W , Chauhan AK , Wagner DD . Glycoprotein Ibα and von Willebrand factor in primary platelet adhesion and thrombus formation: lessons from mutant mice. Thromb Haemost. 2008;99:264–70. 18278173 10.1160/TH07-10-0638

[42] De Meyer SF , Savchenko AS , Haas MS , Schatzberg D , Carroll MC , Schiviz A , et al. Protective anti‐inflammatory effect of ADAMTS13 on myocardial ischemia/reperfusion injury in mice. Blood. 2012;120:5217–23. 22915644 10.1182/blood-2012-06-439935 PMC3537313

[43] Masias C , Cataland SR . Novel therapies in thrombotic thrombocytopenoc purpura. Res Pract Thromb Haemost. 2018;2:19–26. 30046703 10.1002/rth2.12066 PMC6055500

[44] Wise RJ , Dorner AJ , Krane M , Pittman DD , Kaufman RJ . The role of von Willebrand factor multimers and propeptide cleavage in binding and stabilization of factor VIII. J Biol Chem. 1991;266:21948–55. 1939217

[45] Liu Y , Starr MD , Bulusu A , Pang H , Wong NS , Honeycutt W , et al. Correlation of angiogenic biomarker signatures with clinical outcomes in metastatic colorectal cancer patients receiving capecitabine, oxaliplatin, and bevacizumab. Cancer Med. 2013;2:234–42. 23634291 10.1002/cam4.71 PMC3639662

[46] Sweeney JD , Killion KM , Pruet CF , Spaulding MB . von Willebrand factor in head and neck cancer. Cancer. 1990;66:2387–889. 2245394 10.1002/1097-0142(19901201)66:11<2387::aid-cncr2820661123>3.0.co;2-u

[47] Hivert B , Caron C , Petit S , Charpy C , Fankam‐Siaka C , Lecocq S , et al. Clinical and prognostic implications of low or high level of von Willebrand factor in patients with Waldenstrom macroglobulinemia. Blood. 2012;120:3214–21. 22896002 10.1182/blood-2011-11-388256

[48] Terraube V , Pendu R , Baruch D , Gebbink MF , Meyer D , Lenting PJ , et al. Increased metastatic potential of tumor cells in von Willebrand factor‐deficient mice. J Thromb Haemost. 2006;4:519–26. 16405520 10.1111/j.1538-7836.2005.01770.x

[49] Claus RA , Bockmeyer CL , Budde U , Kentouche K , Sossdorf M , Hilberg T , et al. Variations in the ratio between von Willebrand factor and its cleaving protease during systemic inflammation and association with severity and prognosis of organ failure. Thromb Haemost. 2009;101:239–47. 19190805

[50] Karim F , Adil SN , Afaq B , Ul Haq A . Deficiency of ADAMTS‐13 in pediatric patients with severe sepsis and impact on in‐hospital mortality. BMC Pediatr. 2013;13:44. 23537039 10.1186/1471-2431-13-44 PMC3637410

[51] Fukushima H , Nishio K , Asai H , Watanabe T , Seki T , Matsui H , et al. Ratio of von Willebrand factor propeptide to ADAMTS13 is associated with severity of sepsis. Shock. 2013;39:409–14. 23481506 10.1097/SHK.0b013e3182908ea7

[52] Peigne V , Azoulay E , Coquet I , Mariotte E , Darmon M , Legendre P , et al. The prognostic value of ADAMTS13 (a disintegrin and metalloprotease with thrombospondin type 1 repeats, member 13) deficiency in septic shock patients involves interleukin‐6 and is not dependent on disseminated intravascular coagulation. Crit Care. 2013;17:R273. 24238574 10.1186/cc13115 PMC4056532

[53] Hyseni A , Kemperman H , de Lange DW , Kesecioglu J , de Groot PG , Roest M . Active von Willebrand factor predicts 28‐day mortality in patients with systemic inflammatory response syndrome. Blood. 2014;123:2153–6. 24458436 10.1182/blood-2013-08-508093

[54] Martin K , Borgel D , Lerolle N , Feys HB , Trinquart L , Vanhoorelbeke K , et al. Decreased ADAMTS‐13 (A disintegrin‐like and metalloprotease with thrombospondin type 1 repeats) is associated with a poor prognosis in sepsis‐induced organ failure. Crit Care Med. 2007;35:2375–82. 17944029 10.1097/01.ccm.0000284508.05247.b3

[55] Hyun J , Kim HK , Kim JE , Lim MG , Jung JS , Park S , et al. Correlation between plasma activity of ADAMTS‐13 and coagulopathy, and prognosis in disseminated intravascular coagulation. Thromb Res. 2009;124:75–9. 19162307 10.1016/j.thromres.2008.11.020

[56] Habe K , Wada H , Ito‐Habe N , Hatada T , Matsumoto T , Ohishi K , et al. Plasma ADAMTS13, von Willebrand factor (VWF) and VWF propeptide profiles in patients with DIC and related diseases. Thromb Res. 2012;129:598–602. 22070827 10.1016/j.thromres.2011.10.011

[57] Djamiatun K , van der Ven AJ , de Groot PG , Faradz SMH , Hapsari D , Dolmans WM , et al. Severe dengue is associated with consumption of von Willebrand factor and its cleaving enzyme ADAMTS‐13. PLoS Negl Trop Dis. 2012;6:1–8. 10.1371/journal.pntd.0001628 PMC3341341 22563509

[58] Reiter RA , Knobl P , Varadi K , Turecek PL . Changes in von Willebrand factor‐cleaving protease (ADAMTS13) activity after infusion of desmopressin. Blood. 2003;101:946–8. 12393734 10.1182/blood-2002-03-0814

[59] Geisen U , Zieger B , Nakamura L , Weis A , Heinz J , Michiels JJ , et al. Comparison of von Willebrand factor (VWF) activity VWF: Ac with VWF ristocetin cofactor activity VWF:RCo. Thromb Res. 2014;134:246–50. 24891215 10.1016/j.thromres.2014.04.033

